# Melatonin enhances captopril mediated cardioprotective effects and improves mitochondrial dynamics in male Wistar rats with chronic heart failure

**DOI:** 10.1038/s41598-023-50730-z

**Published:** 2024-01-05

**Authors:** Sherein F. El-Sayed, Amira Mohamed Abdelhamid, Shimaa Gamal ZeinElabdeen, Dalia Ibrahim El-wafaey, Suzan M. M. Moursi

**Affiliations:** 1 Medical Physiology Department, Faculty of Medicine, Zagazig University, Zagazig, Egypt; 2 Clinical Pharmacology Department, Faculty of Medicine, Zagazig University, Zagazig, Egypt; 3 Cardiology Department, Faculty of Medicine, Zagazig University, Zagazig, Egypt; 4 Human Anatomy and Embryology Department, Faculty of Medicine, Zagazig University, Zagazig, Egypt

**Keywords:** Cell biology, Cardiology, Health care, Medical research

## Abstract

Mitochondrial dysfunction is a recent emerging research scope that proved to be involved in many cardiovascular diseases culminating in chronic heart failure (CHF), which remains one of the primary causes of morbidity and mortality. This study investigated the added cardio-protective effects of exogenous melatonin administration to conventional captopril therapy in isoproterenol (ISO) exposed rats with CHF. Five groups of Wistar rats were recruited; (I): Control group, (II): (ISO group), (III): (ISO + captopril group), (IV): (ISO + melatonin group) and (V): (ISO + melatonin/captopril group). Cardiac function parameters and some oxidant, inflammatory and fibrotic markers were investigated. Moreover; mRNA expression of mitochondrial mitophagy [parkin & PTEN induced kinase 1 (PINK1)], biogenesis [Peroxisome proliferator-activated receptor gamma coactivator 1-alpha (PGC-1α)], fusion [mitofusin 2 (Mfn2)] and fission [dynamin-related protein 1 (DRP-1)] parameters in rat’s myocardium were evaluated. Rats’ myocardium was histo-pathologically and immunohistochemically evaluated for Beclin1 and Sirt3 expression. The present study revealed that captopril and melatonin ameliorated cardiac injury, oxidative stress biomarkers, and pro-inflammatory cytokines in ISO-exposed rats. These protective effects could be attributed to mitochondrial dynamic proteins control (i.e. enhanced the mRNA expression of parkin, PINK1, PGC-1α and Mfn2, while reduced DRP-1 mRNA expression). Also, Beclin1 and Sirt3 cardiac immunoreactivity were improved. Combined captopril and melatonin therapy showed a better response than either agent alone. Melatonin enhanced myocardial mitochondrial dynamics and Sirt3 expression in CHF rats and may represent a promising upcoming therapy added to conventional heart failure treatment.

## Introduction

Despite significant advancements in diagnosis and treatment, heart failure remains one of the world's major causes of morbidity and mortality^1^. Increased sympathetic excitability, aberrant humoral factor production, overexpression of inflammatory cytokines, imbalance of the immune system and cardiac interstitial fibrosis, are all important aspects of chronic heart failure (CHF) complex pathophysiology^2,3^.

Compared to other tissues, heart tissue contains an abundance of mitochondria, which comprise roughly 45% of the myocardial volume^4^. This abundance results from the cardiomyocytes' high energy requirements, which use more than 90% of the ATP produced by mitochondria^5^.

Lately, numerous dynamic mechanisms controlling mitochondrial function in mammals have been discovered. Fission/fusion, selective mitochondrial autophagy (mitophagy), and mitochondrial biogenesis are some of these mitochondrial dynamics (regulating mitochondrial number for cellular adaptation). Energy generation is regulated by mitochondrial dynamics, which is a balance between mitochondrial fusion and fission^6^.

Moreover, maintaining mitochondrial function and preventing cell death are both dependent on mitophagy, a selective form of autophagy for the targeted degradation of damaged mitochondria^7^. Defective mitochondria and dangerous reactive oxygen species (ROS), which can cross the plasma membrane, may accumulate as a result of damaged mitophagy^8^.

Several cardiac diseases are linked to mitochondrial dysfunction and excessive production of reactive oxygen species (ROS) which damage cellular lipids, proteins, enzymes, and DNA and are associated with apoptosis which accelerates cardiovascular damage^6^.

In addition, the upregulated renin–angiotensin–aldosterone system increases the production of reactive oxygen species (ROS), which causes an inflammatory state and myocyte degeneration in CHF^9^. Hence, angiotensin-converting enzyme inhibitors (ACE-I) are established therapy in patients with heart failure^10^. However, little literature investigated their effect on mitochondrial dynamic parameters.

Melatonin, an endogenous hormone released by the pineal gland has been handled in several studies as a cardioprotective agent in multiple heart diseases, such as atherosclerosis, hypertension, MI/R injury and heart failure as well as has shown to be critical in mitophagy and other mitochondrial activities^11,12^. Melatonin can traverse all cell membranes and collect in subcellular parts, especially mitochondria, due to its amphiphilic nature and tiny size^13^ where it counteracts lipid peroxidation and DNA injury^14^.

So, this study aims to evaluate the effects of both captopril and melatonin on cardiovascular remodelling and function in isoproterenol-exposed rats with CHF and evaluate their effects on mitochondrial dynamics and mitophagy.

Cytosolic Beclin 1 was evaluated as a marker of autophagy. Mitochondrial PTEN-induced kinase 1 (PINK1) and Parkin mRNA expression were assessed as markers of mitophagy. For assessment of mitochondrial biogenesis, the expression of cytosolic Peroxisome proliferator-activated receptor gamma coactivator 1-alpha (PGC-1α) was evaluated. Mitofusin2 (Mfn2) related mitochondrial fusion and [dynamin-related protein 1 (DRP-1) mediated mitochondrial fission was also investigated in response to added melatonin and captopril therapy.

## Material and methods

### Animals

50 healthy adult male Wistar rats (8–10 weeks old and 208–235 gm) obtained from the animal house of the Faculty of Veterinary Medicine—Zagazig University, were kept in hygienic conditions and received a commercial diet and ad libitum water, kept at a temperature of 24 ± 6 °C and on a 12 h light/dark cycle. The study protocol was approved by the Ethics of the guiding standard for the use of research animals by the ZU-IACUC committee, with the number ZU-IACUC/3/F/77/2022. Our study followed the ARRIVE and international ethical guidelines for the care and use of laboratory animals.

### Drugs

Melatonin (5 mg tablet, Natrol Company, USA) and captopril (25 mg tablet, Smithkline Beecham, Giza, Egypt) were purchased. Isoproterenol hydrochloride powder was obtained from Sigma-Aldrich, Cairo, Egypt. Thiopental Sodium (farcopental 0.5 gm/vial, Powder) was obtained from Sigmatec Pharmaceutical Industries, Egypt.

### Experimental design

Rats were randomly assigned to five equal groups: Group I (Control); rats were subcutaneously (S.C) injected with 5 ml/kg physiological saline (0.9%) once daily for 10 successive days, group II (ISO); isoproterenol hydrochloride, dissolved in physiological saline, was S.C injected (5 mg/kg body weight once daily for 10 days) to establish cardiac remodelling^15^, group III (ISO + captopril): Rats were treated with isoproterenol hydrochloride as group II with concurrent captopril administration at a dose of 13.5 mg/kg/day by gavage for 14 days starting from 1st day of ISO administration^16^, group IV (ISO + melatonin)): isoproterenol hydrochloride as group II was administered concurrently with melatonin at a dose of 10 mg/kg/day by gavage for 14 days starting from 1st day of ISO administration^17^, Group V (ISO + melatonin/captopril): isoproterenol was administered concurrently with both melatonin and captopril at the same doses and durations mentioned before.

### Measurement of systolic blood pressure (SBP) and heart weight index (HWI)

SBP was measured on the 1st, 7th, and 14th days of the experiment in millimetres mercury (mm Hg) by a Non-Invasive Blood Pressure Monitor (NIBP 250, Serial No.: 21202-108, BIOPAC System, Inc.; USA)^18^. HWI was measured by dividing the heart weight by body weight.

### Echocardiographic assessment

Echocardiographic examination was performed on day 14 to confirm cardiac remodeling and failure, using a VIVID 3 Ultrasound and 12 MHZ linear transducer and 5–8 MHZ sector transduced. Rats were anaesthetized with 100 mg/kg/i.p ketamine and the ultrasound probe was placed in the left sternal border after shaving their chest hair. Measurements of left ventricular diastolic dimension (LVDD), left ventricular systolic dimension (LVSD) and Ejection fraction were calculated from the M-mode at two-dimensional images obtained in the left parasternal long and short axes at the level of the papillary muscles after observation of at least 6 cardiac cycles.

### Blood sample collection and preparation of tissue homogenate

Rats were anaesthetized with intraperitoneal thiopental (15 mg/kg) after overnight fasting and blood samples were collected from retro-orbital venous plexus, and serum was separated by centrifugation at 3000 rpm for 20 min and kept deep frozen (− 20 °C) until used to measure the serum levels of myocardial injury markers; lactate dehydrogenase (LDH), creatine phosphokinase muscle/brain (CPK-MB), cardiac troponin I (cTnI) by commercially available kits (Bayer Diagnostics Ltd., Baroda, India for LDH, BioMérieux Diagnostics, Milan, Italy for CK-MB and Sangon Biotech Co., Ltd., Shanghai, China for cTnI) according to the manufacturer’s instructions. Then animals were sacrificed, and hearts were excised immediately, washed with saline, then weighed and divided into two portions. A portion of the cardiac tissue was preserved immediately in liquid nitrogen, then homogenized with phosphate buffered solution (PBS, 7.4 pH), and then centrifuged at 12,000×*g* for 10 min at 4 °C, the supernatant portion was used for measuring cardiac biochemical parameters^19^. The other portion was rinsed in 10% formaldehyde solution and preserved for histopathological and immunohistochemical examination.

### Measurement of lipid peroxidation and antioxidant status

Malondialdehyde (MDA) and superoxide dismutase (SOD) assay kits were used (Bio diagnostic, Egypt kits) according to their manufacturers’ protocols.

### Determination of cardiac inflammatory/anti-inflammatory, apoptotic, and fibrotic markers (TNF-α, IL-6, IL-10, iNOS, caspase-3, TGF-β1, and collagen-I)

Rat ELISA kits for TNF-α, IL-6 and IL-10 (Bio diagnostic, Egypt kits), caspase-3 (CUSABIO, Baltimore, Maryland, USA) and TGF-β1, collagen-I and iNOS (Sigma-Aldrich Co., Egypt) were used to measure these parameters according to their manufacturers’ protocols.

### Histopathological analysis and immunohistochemical (IHC) study for Beclin-1 and Sirt3 expression

The remaining cardiac tissue was fixed in 10% buffered formalin and embedded in paraffin. Thick sections of 4–5 um were cut from the tissue block, mounted on glass slides then deparaffinized in xylene, and stained with haematoxylin and eosin (H & E) and Masson’s Trichome (MT) stains to observe collagen fibres deposits.

Serial sections (4–5 μ) cut from the paraffin blocks were boiled in 10 mM citrate buffer (AP9003) at pH 6 for 10 min to retrieve antigen, then incubated for 1 h with the primary antibodies of an anti-Beclin-1 antibody (ab217179) (rabbit anti-human polyclonal antibody, dilution 1:150, Abcam) and polyclonal Sirt3 (cat. no. sc-15404; Santa Cruz Biotechnology). After the automatic immunostainer (DAKO autostainer), IHC analysis was performed using the polymer Envision detection system; the Dako EnVision ™ kit (Dako, Copenhagen, Denmark). Finally, the sections were contrasted with Mayer’s haematoxylin at 0.1%. Analysis was performed by a blinded pathologist.

### Morphometric study

Image J analysis software was used (Fiji image j; 1.51 n, NIH, USA) within 10 non-overlapping fields for each section. The area percent of collagen fibres and Beclin-1 & Sirt3 Immunoreaction were measured after light microscope magnification at 10× and 40× respectively.

### Quantitative RT-PCR

Total RNA was extracted from rats’ homogenized myocardium using Trizol (Thermo Fisher Scientific, Waltham, MA, USA) and reversely transcribed to cDNA using a Revert Aid First Strand cDNA Synthesis kit (Fermentas, Vilnius, Lithuania). The cDNA was subjected to quantitative RT-PCR implemented in a StepOnePlus Real-Time PCR system (Applied Biosystems, USA) using iQ SYBR Green supermix (BioRad Laboratories, Hercules, CA, USA). The primer sequences (Thermo Fischer Scientific, USA) were as follows:

**Table:** 

	Forward	Reverse
Parkin	5′-CTGGCAGTCATTCTGGACAC-3′	5′-CTCTCCACTCATCCGGTTTG)-3′
Pink1	5′-CATGGCTTTGGATGGAGAGT-3′	5′-TGGGAGTTTGCTCTTCAAGG-3′
PGC-1α	5′-CGCAACATGCTCAAGCCAAA-3′	5′-GCGGTCTCTCAGTTCTGTCC-3′
Mfn2	5′-ATGCATCCCCACTTAAGCAC-3′	5′-CCAGAGGGCAGAACTTTGTC-3
DRP1	5′-CACCCGGAGACCTCTCATTC-3′	5′-CCCCATTCTTCTGCTTCCAC-3′
GAPDH	5′-GGCACAGTCAAGGCTGAGAATG-3′	5′-ATGGTGGTGAAGACGCCAGTA-3′

The level of each gene expression was normalized to the level of the housekeeping gene, Gene—Glyceraldehyde-3-Phosphate Dehydrogenase (GAPDH). The results were detected as fold change by the 2^–ΔΔCT^ method.

### Statistical analysis

The SPSS program (version 20) was used to statistically evaluate the data, which were reported as mean ± SD (SPSS Inc. Chicago, IL, USA). One-way analysis of variance (ANOVA) was performed to assess group differences, and the student's least significant differences (LSD) test was applied for post hoc comparisons. Statistics were deemed significant at *p* < 0.05.

### Ethics approval

The ZU-IACUC committee's Ethics of the guiding standard for the use of research animals accepted the study protocol, with number ZU-IACUC/3/F/77/2022.

## Results

### Effect of captopril and/ or melatonin on systolic blood pressure (SBP)

**Figure 1:**
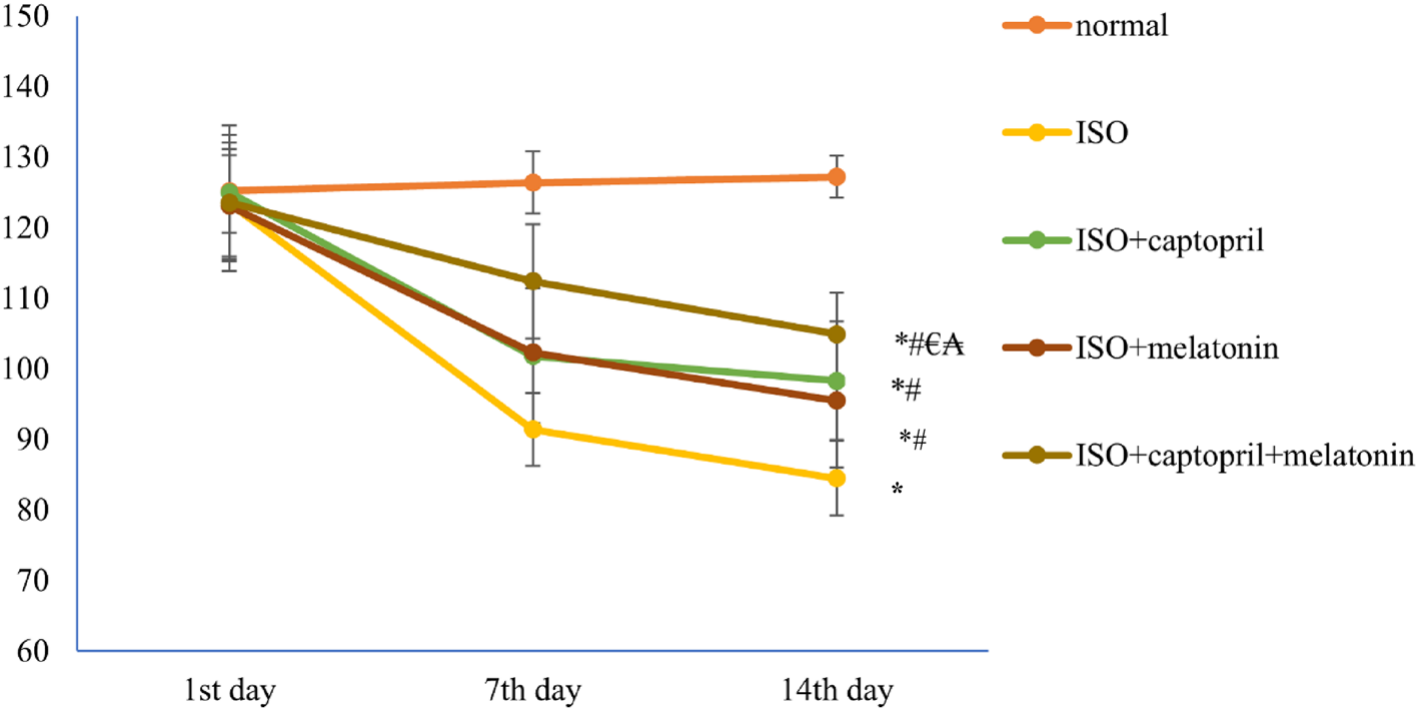
Effects of captopril, melatonin, and their combination on systolic blood pressure (SBP). *p < 0.01 versus control, ^#^*p* < 0.01 versus (ISO) group, ^€^*p* < 0.01 versus ISO + captopril group and ^₳^*p* < 0.01 versus ISO + melatonin group.

Compared to the normal control group, SBP was significantly reduced in the ISO group (*p* < 0.001). It was improved and increased in the treated captopril and melatonin groups when compared to the ISO untreated group (*p* < 0.001). Moreover, their combination therapy in the 5th group showed more significant SBP improvement than either drug alone (*p* < 0.01)] (Fig. 1).

### Effect of captopril and/or melatonin on heart weight, heart weight index (HWI) and cardiac function and morphological alterations

**Figure 2:**
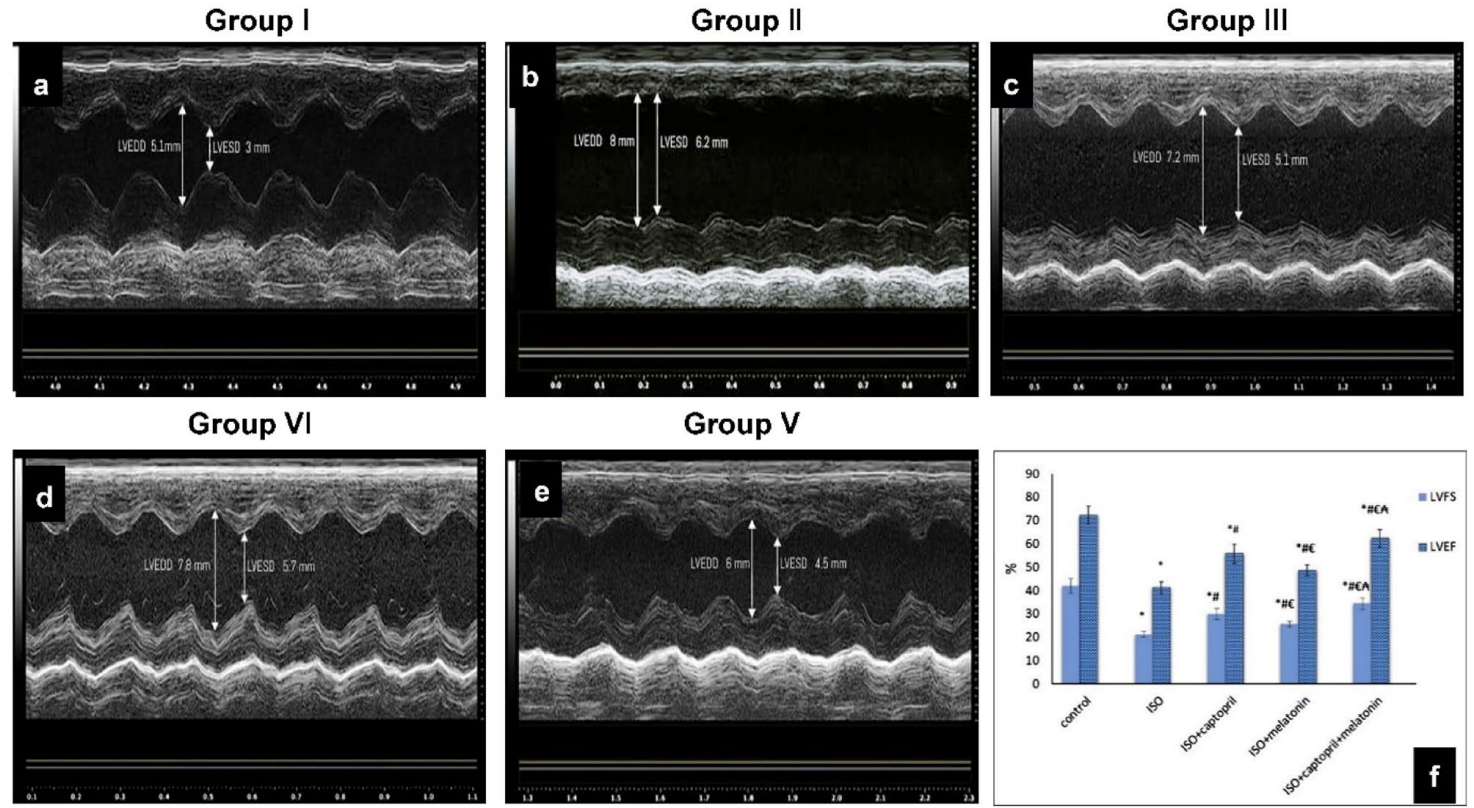
Echocardiographic views for control (**a**), (ISO) (**b**), ISO + captopril (**c**) ISO + melatonin (**d**) and ISO + melatonin/captopril (**e**). Statistical analysis showing the percentage of left ventricular ejection fraction (LVEF) and left ventricular fractional shortening (LVFS) in all groups (**f**). *p < 0.001 versus control, ^#^*p* < 0.001 versus (ISO) group, ^€^*p* < 0.001 versus ISO + captopril group and ^₳^*p* < 0.001 versus ISO + melatonin group.

Echocardiography revealed an increase in left ventricular dimensions, (LVESD and LVEDD), while, LVFS and LVEF were significantly decreased in the ISO group in comparison to the normal control group (*p* < 0.001). Also, the heart weight and heart weight index were increased in the ISO group. However, all these previous changes were significantly improved (LVESD, LVEDD, heart weight and heart weight index showed a significant decrease and LVFS and LVEF showed a significant increase) in treated groups and the most significant improvement was recorded in the combined therapy (group V) (Table 1, Fig. 2).

**Table 1:** Weights and Echocardiographic parameters in all studied groups.

Groups						
Parameters		Group I (control)	Group II (ISO)	Group III (ISO + captopril)	Group IV (ISO + melatonin)	Group V (ISO + melatonin + captopril)
Body weight (g)	Mean ± SD	229.8 ± 10.2	219.1 ± 10.8	228.8 ± 8.6	222.2 ± 10.5	228.1 ± 8.8
	*p* value		*p* > 0.05^a^	*p* > 0.05^a,b^	*p* > 0.05^a,b,c^	*p* > 0.05^a,b,c,d^
Heart weight (g)	Mean ± SD	1.12 ± 0.09	1.73 ± 0.11	1.43 ± 0.11	1.57 ± 0.08	1.28 ± 0.06
	*p* value		*p* < 0.001^a^	*p* < 0.001^a,b^	*p* < 0.001^a,b^	*p* < 0.001^a,b,d^
					*p* < 0.01^c^	*p* < 0.01^c^
HWI (mg/g)	Mean ± SD	4.89 ± 0.6	7.9 ± 0.43	6.24 ± 0.37	7.06 ± 0.24	5.61 ± 0.16
	*p* value		*p* < 0.001^a^	*p* < 0.001^a,b^	*p* < 0.001^a,b,c^	*p* < 0.001^a,b,c,d^
LVEDD (mm)	Mean ± SD	5.33 ± 0.42	8.92 ± 0.63	7.14 ± 0.56	8.04 ± 0.55	6.26 ± 0.53
	*p* value		*p* < 0.001^a^	*p* < 0.001^a,b^	*p* < 0.001^a,b,c^	*p* < 0.01^a,b,c,d^
LVESD (mm)	Mean ± SD	3.1 ± 0.40	7.03 ± 0.6	5.01 ± 0.56	5.99 ± 0.51	4.11 ± 0.47
	*p* value		*p* < 0.001^a^	*p* < 0.001^a,b^	*p* < 0.001^a,b,c^	*p* < 0.001^a,b,c,d^
LVFS %	Mean ± SD	42 ± 3.12	21.3 ± 1.33	30 ± 2.44	25.6 ± 1.34	34.4 ± 2.45
	*p* value		*p* < 0.001^a^	*p* < 0.001^a,^	*p* < 0.001^a,b,c^	*p* < 0.001^a,b,c,d^
LVEF %	Mean ± SD	72.4 ± 3.83	41.3 ± 2.4	55.9 ± 4.06	48.7 ± 2.4	62.4 ± 3.65
	*p* value		*p* < 0.001^a^	*p* < 0.001^a,b^	*p* < 0.001^a,b,c^	*p* < 0.001^a,b,c,d^

SD (standard deviation), a = versus group I, b = versus group II, c = versus group III; d = versus group IV. (HWI) heart weight index, LVEDD; left ventricular End diastolic dimension, LVESD; left ventricular End systolic dimension, LVFS; left ventricular fractional shortening, LVEF; left ventricular ejection fraction.

### Effect of captopril and/or melatonin on serum cardiac injury markers

**Figure 3:**
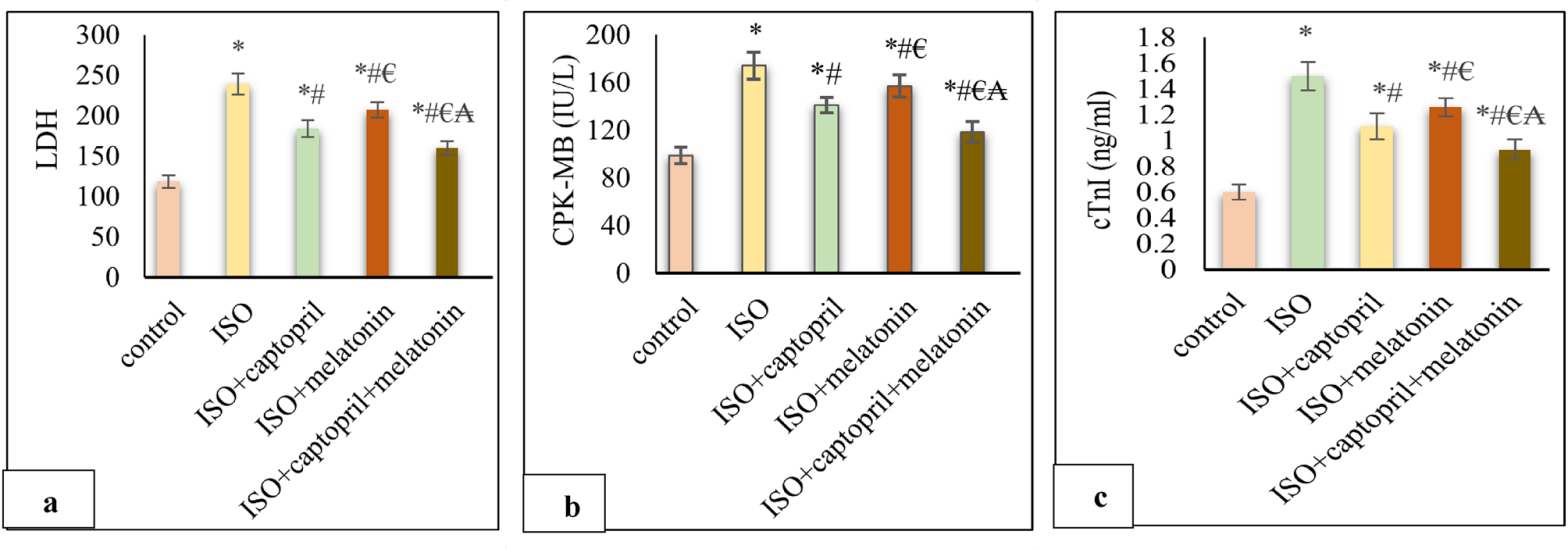
Effects of captopril, melatonin, and their combination on serum cardiac injury markers; LDH (**a**), (CPK-MB) (**b**) and (cTnI) (**c**). *p < 0.001 versus control, ^#^*p* < 0.001 versus (ISO) group, ^€^*p* < 0.001 versus ISO + captopril group and ^₳^*p* < 0.001 versus ISO + melatonin group.

The levels of serum LDH, CPK-MB and cTnI showed a significant increase in the ISO group compared to the control group (*p* < 0.001) which were significantly improved by melatonin and/ or captopril administration. Notably, the combined therapy in group V showed the best improvement than either drug alone (*p* < 0.001) (Fig. 3).

### Effect of captopril and/or melatonin on cardiac oxidant/antioxidant markers

**Figure 4:**
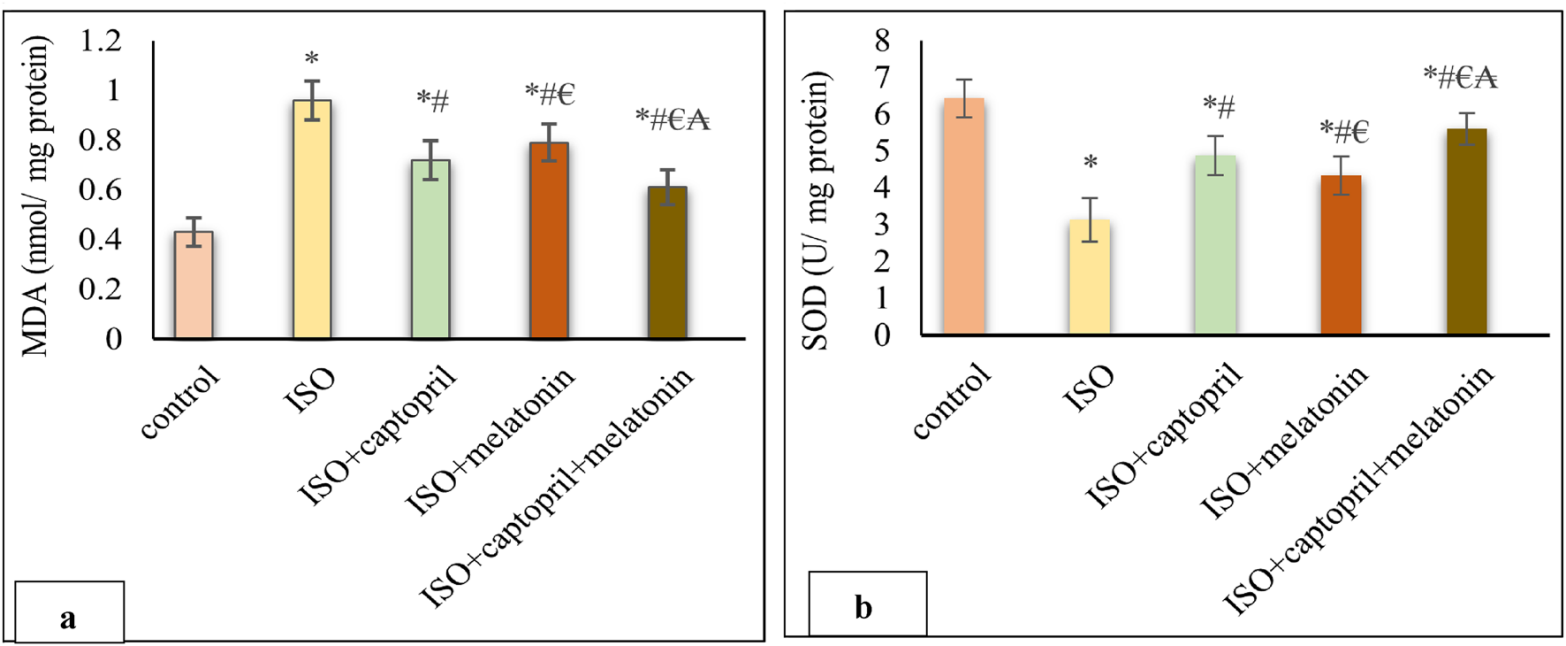
Effects of captopril, melatonin, and their combination on cardiac oxidant/antioxidant markers; MDA (**a**) and (SOD) (**b**). *p < 0.001 versus control, ^#^*p* < 0.001 versus (ISO) group, ^€^*p* < 0.001 versus ISO + captopril group and ^₳^*p* < 0.001 versus ISO + melatonin group.

Cardiac MDA was significantly increased; however cardiac SOD activity was significantly decreased in the ISO group compared to the control group (*p* < 0.001). These effects of ISO were significantly mitigated with, melatonin and/ or captopril. Of note, the combination therapy showed a more significant improvement compared with that of monotherapy (*p* < 0.01) (Fig. 4).

### Effect of captopril and/or melatonin on cardiac inflammatory/anti-inflammatory cytokines

**Figure 5:**
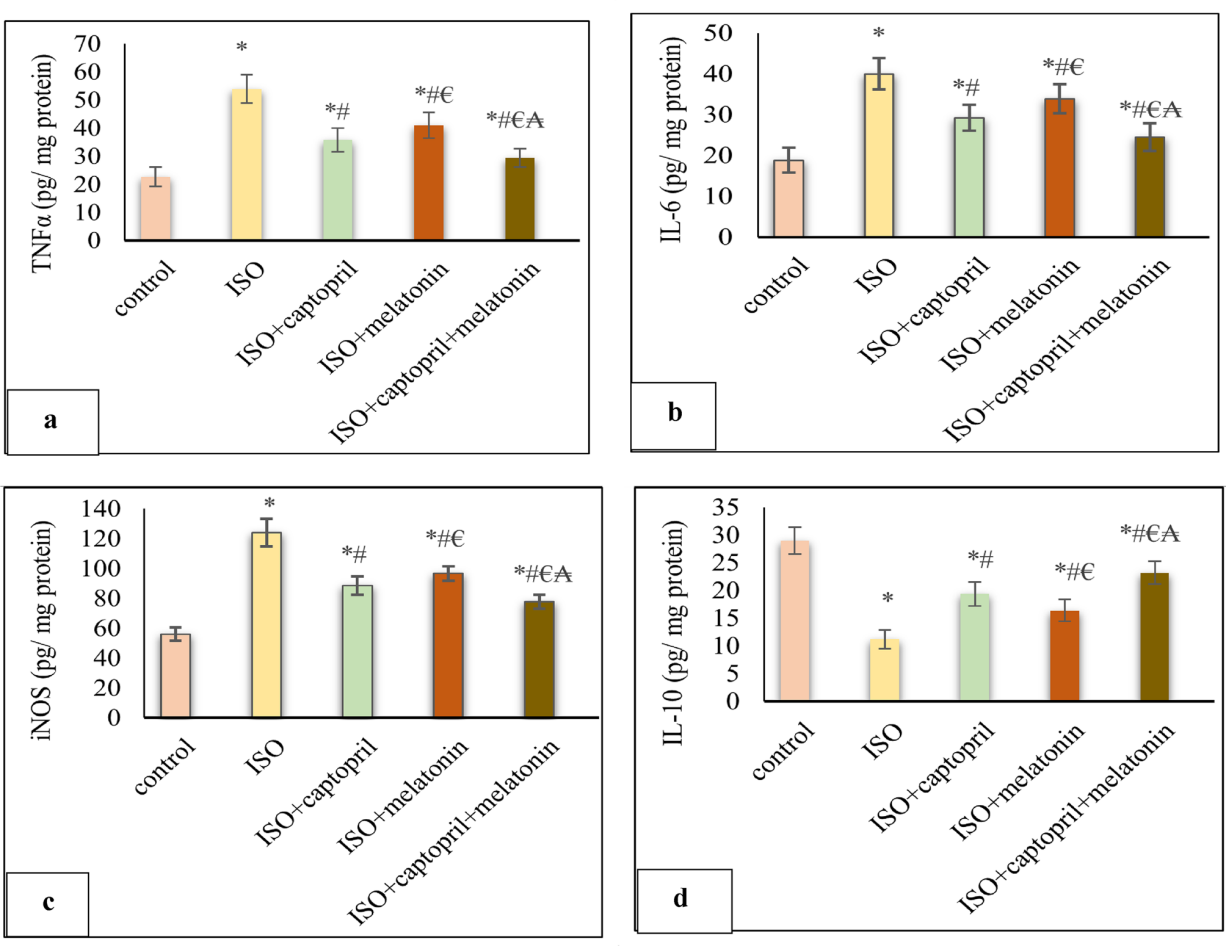
Effects of captopril, melatonin, and their combination on cardiac inflammatory/ anti-inflammatory cytokines; (TNF-α) (**a**), (IL-6) (**b**), (iNOS) (**c**) and (IL-10) (**d**). *p < 0.001 versus control, ^#^*p* < 0.001 versus (ISO) group, ^€^*p* < 0.001 versus ISO + captopril group and ^₳^*p* < 0.001 versus ISO + melatonin group.

Cardiac TNF-α, IL-6 as well and iNOS were significantly increased, however cardiac IL-10 level was significantly decreased in the ISO group, compared to the control group (*p* < 0.001). Melatonin and/ or captopril significantly ameliorated these changes with better improvement observed at their combination (Fig. 5).

### Effect of captopril and/or melatonin on cardiac apoptotic marker caspase 3 and fibrotic markers

**Figure 6:**
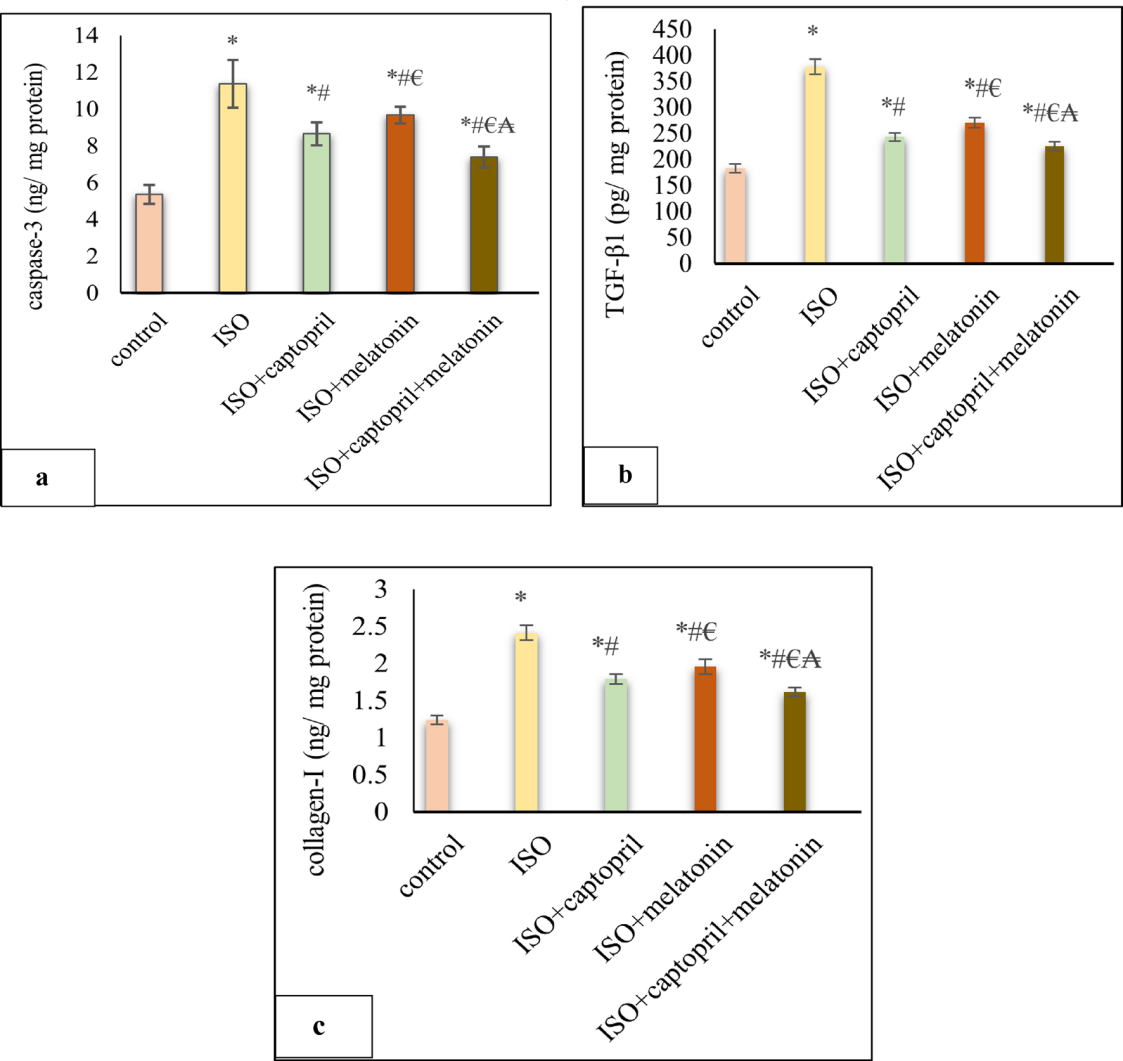
Effects of captopril, melatonin, and their combination on cardiac apoptotic marker caspase 3(**a**) and fibrotic markers (TGF-β1) (**b**) and collagen-I (**c**). *p < 0.001 versus control, ^#^*p* < 0.001versus (ISO) group, ^€^*p* < 0.001versus ISO + captopril group and ^₳^*p* < 0.001 versus ISO + melatonin group.

Cardiac caspase-3 was significantly increased in the ISO group compared to the control (*p* < 0.001). On the other hand, significantly decreased with melatonin and/ or captopril compared to the ISO-untreated group (*p* < 0.001). Concurrent administration of captopril and melatonin showed more significant effects (*p* < 0.001) (Fig. 6). In addition, the ISO untreated group showed a significant increase in TGF-β1 and collagen-I cardiac tissue levels when compared to the control group (*p* < 0.001). However, melatonin and/ or captopril significantly (*p* < 0.001) suppressed this increase which was the best with their combination (Fig. 6).

### Effect of captopril and/or melatonin on mRNA expression of mitochondrial dynamics and mitophagy parameters

**Figure 7:**
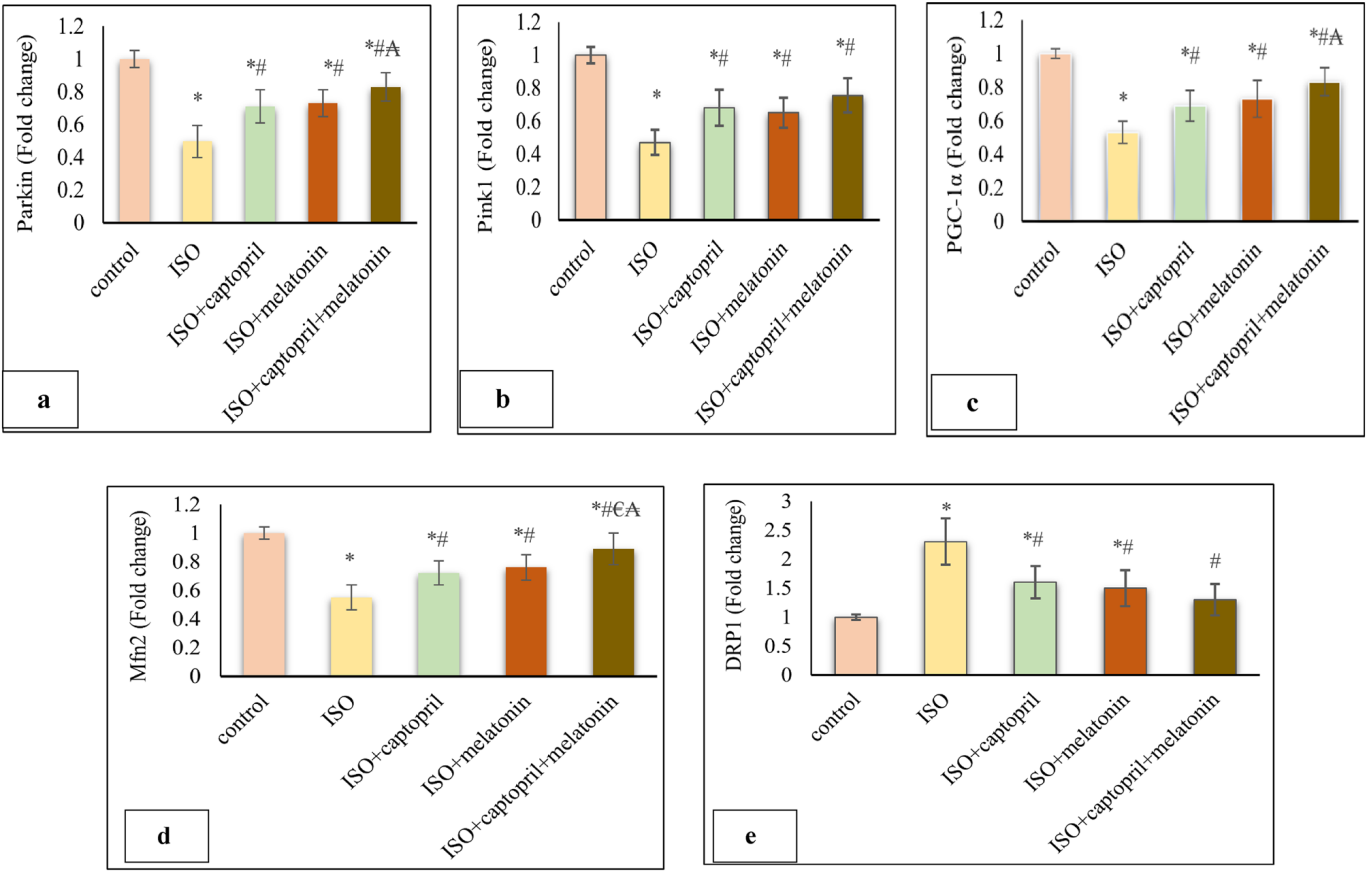
Effects of captopril, melatonin, and their combination on mitochondrial dynamics in the rat model of CHF parkin (**a**) & (PINK1) (**b**), (PGC-1α) (**c**), (Mfn2) (**d**) and (DRP-1) (**e**) parameters in rat’s myocardium. *p < 0.001 versus control, ^#^*p* < 0.001 versus (ISO) group, ^€^*p* < 0.001 versus ISO + captopril group and ^₳^*p* < 0.001 versus ISO + melatonin group.

Our study also revealed significantly impaired mitochondrial dynamics in untreated CHF model; reduced mRNA expression of mitochondrial mitophagy (parkin & PINK1), biogenesis (PGC-1α) and fusion (Mfn2) proteins compared to the control group (*p* < 0.001). Meanwhile, the mRNA expression of the mitochondrial fission Parameter (DRP-1) was significantly increased (*p* < 0.001). Both captopril and/or melatonin alleviated this impairment and significantly restored the mRNA expression of mitochondrial mitophagy (parkin/PINK1), biogenesis (PGC-1α) and fusion (Mfn2) parameters (*p* < 0.001). A significant reduction in the mRNA expression of the mitochondrial fission parameter (DRP-1) was also observed (*p* < 0.001) (Fig. 7). Better improvement was obvious at combination therapy, although sometimes the difference was insignificant.

### Effect of captopril and/or melatonin on myocardial morphology and fibrosis

**Figure 8:**
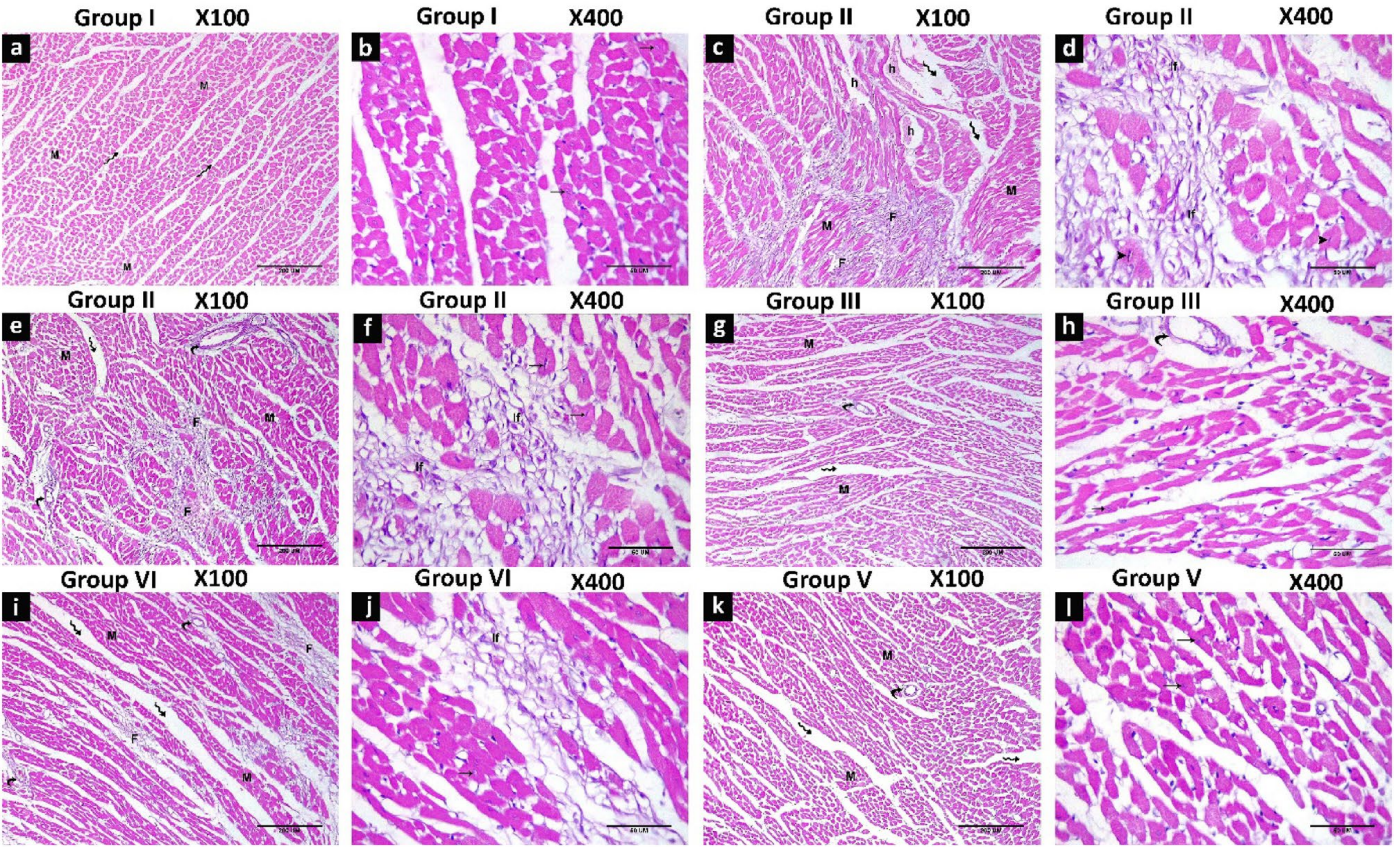
H&E-stained heart tissue sections showing regularly arranged myofibrils (M) with narrow spaces in between in the control group (**a**,**b**). Distorted myofibrils with patches of fibrosis (F), haemorrhage (h) inflammatory cells (If), dilated congested blood vessels with perivascular cuffing (curved arrow) in ISO group (**c**–**f**). Improvement of myofibrils arrangement with wide spaces, dilated and perivascular cuffing in ISO/captopril group (**g**,**h**). Small areas of fibrosis (F), few cellular infiltration (If), perivascular cuffing and wide spaces in the ISO/melatonin group (**i**,**j**). Restoration of the normal arrangement of the myofibrils but areas of wide spaces and perivascular cuffing are still observed in the ISO/captopril/melatonin group (**k**,**l**).

**Figure 9:**
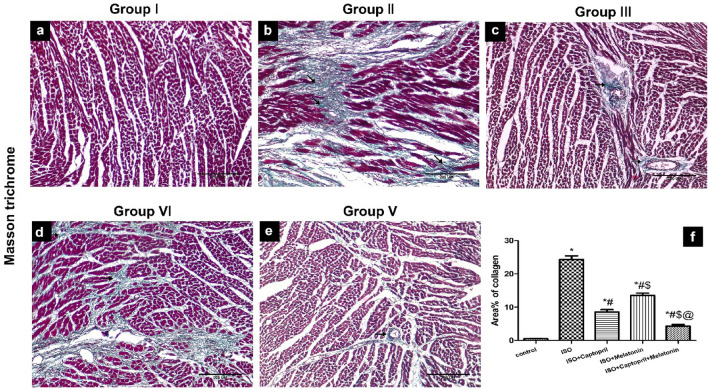
Masson’s trichrome stained heart tissue sections showing collagen fibers (green color). In control group (**a**), wide areas of collagen deposition in the ISO group (**b**), few fibres of collagen around blood vessels in ISO + captopril (**c**) and ISO + melatonin group (**d**) but scanty patches of collagen fibers in noticed in ISO/captopril/melatonin (**e**). Morphometrical analysis shows an increased area percentage of collagen fibres in the ISO group (**f**). Scale bars 3A–3E = 200 µm.

Histopathological examination revealed distorted cardiac muscle fibres with patches of fibrosis, haemorrhage, inflammatory cells and dilated congested blood vessels with perivascular cuffing in ISO group. These manifestations were improved by melatonin and/or captopril administration with a more pronounced effect displayed with their combination (Figs. 8, 9).

### Effect of captopril and/or melatonin on Beclin 1 and Sirt3 expression in myocardial tissue

**Figure 10:**
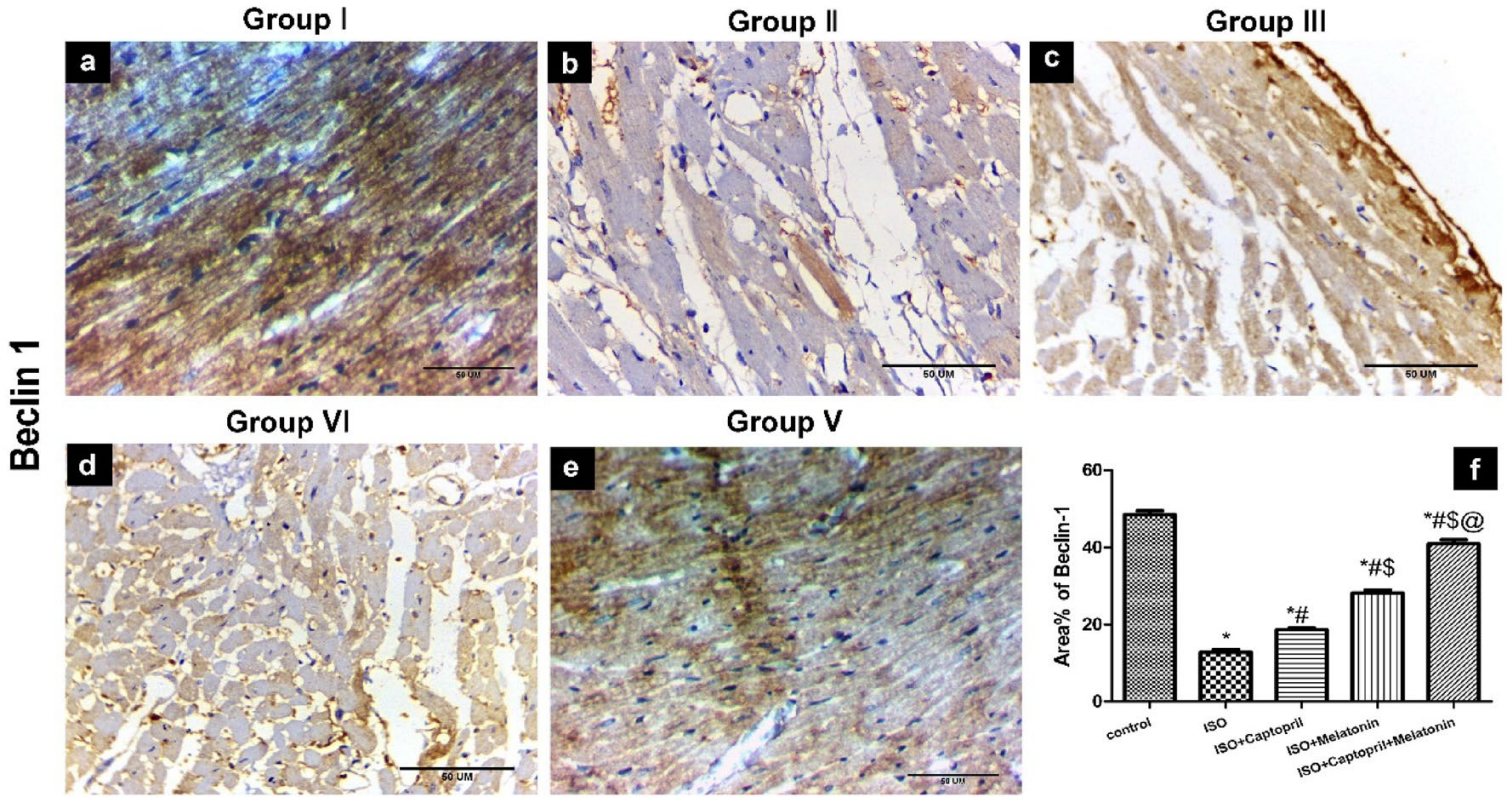
Expression of Beclin 1 in studied groups. The immunohistochemical results showed high positive cytoplasmic immunoreactivity (brown colour) is detected in most of myofibers of the control (**a**) group. The immunoreactivity is seen in a few myofibers of the ISO group (**b**). Positive immunoreactivity in some myofibers in both ISO/captopril (**c**) and ISO/melatonin (**d**) groups. Positive immunoreactivity in many myofibrils in ISO/captopril/melatonin (**e**) group. (**f**) Morphometrical analysis showing a decreased percentage of Beclin-1 immunoreactivity in the ISO group. Scale bars 4A–4E = 50 µm.

**Figure 11:**
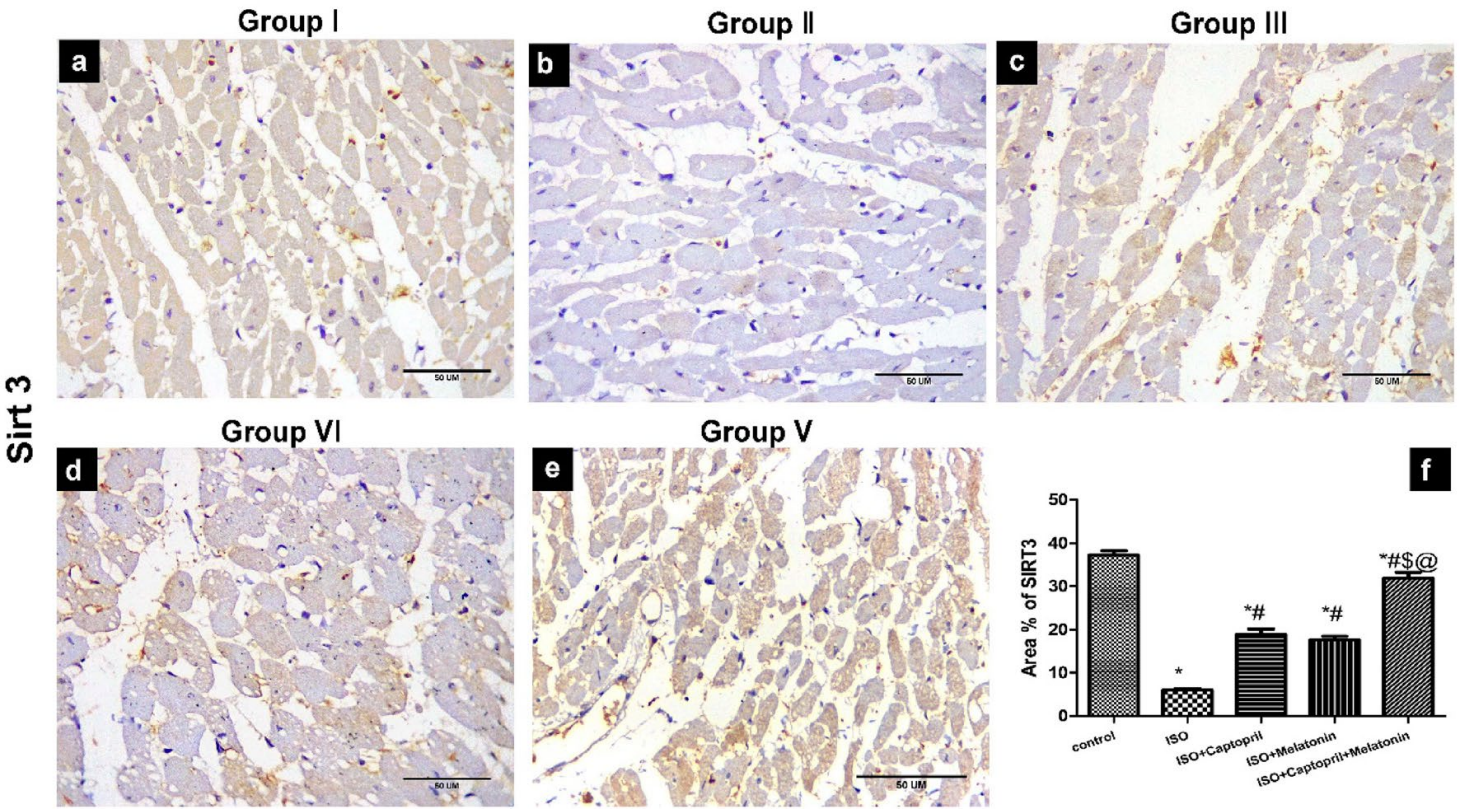
Expression of Sirt3 in studied groups. Sirt3 immuno-stained heart tissue sections showed decreased immunoreactivity (brown colour) of Sirt3 marker in ISO group (**B**) compared to other groups. (**F**) Morphometrical analysis showing a decreased percentage of Sirt3 immunoreactivity in the ISO group. Scale bars 5A–5E = 50 µm.

Our immune-histochemical results also revealed decreased expression of Sirt3 and the mitophagy-related molecule, Beclin1 in ISO group that was abrogated by captopril and/or melatonin administration and similarly, the combined administration gave more significant results (Figs. 10, 11).

## Discussion

The main factor in the development of heart failure and a crucial mechanism in ventricular remodelling is myocardial fibrosis (MF)^16^. When creating cardiovascular therapeutics, new modalities should target the major signalling pathways implicated in cardiac fibrosis^20^.

Many cardiovascular disorders have been linked to disturbed mitochondrial dynamics. Excessive mitochondrial fission is involved in ischemia/reperfusion injury, diabetic cardiomyopathy, and heart failure^21,22^. Mitochondrial fusion insufficiency was also found to be involved in myocarditis, heart failure, and cardiac hypertrophy^23,24^.

This study aimed to investigate the potential benefits of melatonin as a cardioprotective treatment for isoproterenol (ISO) induced chronic myocardial damage in rats, both alone and in combination with captopril and determine the possible underlying mechanisms concerning mitochondrial dynamics.

According to the dose and duration of its application, ISO treatment in rats has been shown to produce cardiac necrosis, hypertrophy, and fibrosis, allowing for the in vivo assessment of anti-fibrotic medicines^25,26^.

The current study demonstrated that isoproterenol administration causes myocardial hypertrophy (as indicated by heart weight and HWI increase) and cardiac dysfunction (as shown by the reduced SBP, LVEF and FS and the increase in cardiac dimensions, LVEDD and LVESD).

Moreover, myocardial histopathological examination showed distorted cardiac muscle fibres associated with exaggerated fibrosis with inflammatory cell infiltration and fibroblastic hyperplasia in the myocardium of the ISO group. Additionally, increased serum concentrations of myocardial enzymes; LDH, CPK-MB and cTnI as clinical markers for myocardial injury were observed. Myocardial enzymes increased in serum proportional to the number of myocardial necrotic cells or injury which alter the plasma membrane integrity and/or permeability.

ISO is a beta-adrenergic receptor (β) agonist that could stimulate both β1 and β2. However, excessive production or exogenous administration induces myocardial ischemia, necrosis, and fibrosis with decreased myocardial compliance, which causes diastolic and systolic dysfunction and closely resembles the alterations found in human heart failure^27^.

We observed in our study that administration of either captopril or melatonin significantly improved SBP, cardiac function and reduced cardiac injury-related protein levels, lessened the effects of ISO on myocardial cellular inflammatory infiltration, myofibrils disarray, extracellular matrix collagen deposition, and hypertrophy. Moreover, the coadministration of both compounds was more efficient in suppressing this damage than either agent alone, confirming their additive cardioprotective role in cardiac remodelling and fibrosis.

Abareshi et al.^28^ also showed that treatment with Captopril, a known ACE inhibitor, has a significant effect on preventing cardiac hypertrophy and left ventricular wall dilation, improved cardiac fibrosis, and lowered collagen content in heart tissue. Captopril has been widely used to alleviate left ventricular dysfunction after myocardial infarction^29^.

Additionally, melatonin has been reported to attenuate ISO-induced cardiac fibrosis and improve the SBP after long-term high-fat diet administration by blocking myocardial ROS production and cell apoptosis^30,31^.

In this study, The ISO-induced oxidative stress (indicated by increased MDA cardiac level as a marker for lipid peroxidation and by reduced SOD activity) was suppressed by captopril or/and melatonin. Several studies showed the protective effect of different anti‐oxidative stress therapies in CHF rat models^32^. Also, they inhibited ISO-induced inflammatory cytokines IL-6, TNF-α and iNOS levels, while IL-10, a cytokine that inhibits inflammation, was markedly elevated. This supports their antioxidants and anti-inflammatory properties reported in previous concordant studies^12,17^. Notably, the combined therapy of captopril and melatonin showed more improvement than either drug alone.

Melatonin has numerous antioxidant effects and due to its lipophilic property, it can easily reach mitochondria and maintain its adequate function by reducing oxidative stress, apoptosis and cell death. Via melatonin receptor, MT3, it acts as a quinone reductase, inhibits electron transfer from quinones and reduces oxidative stress^33^. Melatonin also acts directly as a free radical scavenger, donates electrons, and decreases reactive oxygen and nitrogen species, such as nitric oxide (NO), superoxide anion radical (O^2−^), and hydroxyl radical (OH)^34^. Melatonin via melatonin receptors MT1 and MT2 is also responsible for increasing the levels of antioxidant enzymes including superoxide dismutase, glutathione peroxidase and glutathione reductase, thus decreasing molecular damage^35^.

Similarly, captopril as a thiol-containing molecule confers properties other than ACE inhibition. The SH moiety easily undergoes oxidation and disulfide exchange reactions and is converted into disulfides through the interaction with free radicals. Through this process, it can serve as a free radical scavenger and improve cellular dysfunction. Captopril reduced ROS in several diseased models^36,37^.

Furthermore, the decrease of nuclear factor kappa-light-chain-enhancer of activated B cells (NF-B) activation is another way that melatonin inhibits lipopolysaccharide-stimulated interleukin-1 beta (IL-1), IL-6, TNF-α and iNOS production^12,38^.

Moreover, we observed both captopril and melatonin therapy inhibited ISO-induced cardiomyocyte apoptosis by reducing the expression of caspase-3 and similarly, more improvement was observed in the combined therapy group. Melatonin's ability to inhibit apoptosis and modulate mitochondrial oxidative stress may result from its activation of p-Akt and p-STAT3, which in turn encourages antioxidant gene expression, prevents Bax translocation, increases the expression of the anti-apoptotic protein Bcl2, and inhibits the opening of the mitochondrial permeability transition pore (mPTP), which triggers caspase 3^39^.

Indeed, our results showed that the increased cardiac level of the fibrogenic proteins, TGF-β1, as well as collagen-1 deposits in ISO-exposed myocardium, was significantly reduced by captopril and/or melatonin therapy, thus improving fibrosis and this is concordant to previous studies which reported the melatonin suppressing effect on fibrosis^40^. Melatonin could alleviate myocardial fibrosis via inhibiting TGF-β1/Smads signaling which increases collagen expression and deposition of fibrous tissue^41,42^. Moreover, this fibrosis-reducing effect of melatonin was confirmed in a continuous light-induced hypertension model and while only captopril prevented left ventricular hypertrophy development, only melatonin significantly reduced fibrosis. This antifibrotic action of melatonin may be protective in hypertensive heart disease^43^.

In our ISO-induced CHF model, we observed downregulation of mitochondrial mitophagy, biogenesis and fusion but, simultaneously upregulated mitochondrial fission. Similarly, Mukherjee et al.^44^ and Zhen et al.^45^ revealed that an imbalance between mitochondrial fusion and fission caused by ISO led to an accumulation of non-functional mitochondria and an overabundance of ROS, which in turn caused cell death.

Additionally, as damaged and dysfunctional mitochondria are significant producers of ROS, mitophagy, a particular type of autophagy, is one of the most efficient methods to remove damaged mitochondria, and its impairment is frequently noticed in the development of CHF^46^.

On the other hand, captopril therapy improved the disturbed mitochondrial dynamics in ISO-group by upregulating mitochondrial mitophagy, biogenesis and fusion while downregulating mitochondrial fission. This may be attributed to its reduction of oxidative stress so, protecting mitochondrial membrane potential (MMP) and improving mitochondrial energy production^47,48^.

Similarly, melatonin regained normal mitochondrial dynamics in our model, this is in line with Wang et al.^49^ who demonstrated the promoting effect of melatonin against impaired mitophagy activity by increasing Parkin translocation to the mitochondria in diabetic mice cardiomyopathy. Moreover, Pei et al.^50^ demonstrated that melatonin attenuates post-MI injury by increasing the expression of the mitochondrial fusion protein Mfn2, which is essential for balancing mitochondrial fusion and fission as well as autophagy. Mfn2 is involved in mitochondrial outer membrane fusion^51^. On the contrary, Wu et al.^12^ showed decreased mitophagy-related protein (Parkin and Beclin1) expression and suggested that the cardioprotective benefits of melatonin against anoxia/reoxygenation injury may be due to the suppression of excessive mitophagy. Melatonin modulates mitophagy by boosting or decreasing mitophagic activity, according to studies with various experimental designs^52–55^. These specific regulatory mechanisms need further investigation.

Furthermore, the immunohistochemical results highlighted the protective role of captopril and/or melatonin in their elevation of autophagy-mediator protein Beclin 1 in the heart tissue of treated groups. Autophagy is a conserved catabolic process that plays a critical role in cellular survival by sequestration of cytoplasmic organelles and long-lived proteins into the autophagosome to be degraded by lysosomal enzymes^56^. An important autophagy mediator is beclin-1, and influences both autophagy and apoptosis, thereby deeply affecting cardiomyocytes’ survival and death^57^. Myocardial hypertrophy models used in other studies similarly showed considerably reduced autophagy, which raises the possibility that autophagy might prevent hypertrophy of cardiac muscle^58^.

Moreover, the significant decrease in Sirt3 expression in the myocardium of the ISO group in our model has been reversed by captopril and/or melatonin therapy. Sirt3, a histone deacetylase found in the mitochondria, maintains the body's normal vital functions by balancing energy metabolism, controlling the cell cycle, and protecting against oxidative stress. This prevents the development of myocardial hypertrophy, myocardial fibrosis, and heart failure^59^.

## Conclusion

In summary, combination therapy of melatonin and captopril can rescue chronic heart failure rats and provide an additional benefit to the protection with either agent alone. This is indicated by improved cardiac function, decreased cardiac tissue injury, and ameliorated interstitial fibrosis and hypertrophy. The pharmacologic actions are related to balancing mitochondrial dynamics and alleviating myocardial, inflammation, apoptosis, oxidative stress and fibrosis. Melatonin could be an added strategy for the treatment of heart failure and cardiac fibrosis with captopril.

## Data Availability

All data that support the results in the article are available as mean ± SE values, as well as statistical summaries as supporting information.
